# Case Report: A rare coexistence in children: constrictive pericarditis, atrial septal defect, and cardiac diverticulum

**DOI:** 10.3389/fped.2026.1779452

**Published:** 2026-04-21

**Authors:** Xuan Liu, Qiuying Liu, He Ren, Mei Liu

**Affiliations:** 1 Department of Ultrasound, The Sixth Medical Center of PLA General Hospital, Beijing, China; 2 Department of Pathology, The Sixth Medical Center of PLA General Hospital, Beijing, China

**Keywords:** atrial septal defect, children, constrictive pericarditis, diverticulum, echocardiography

## Abstract

This report describes a rare case of a girl with constrictive pericarditis (CP), coexisting with atrial septal defect (ASD) and cardiac diverticulum. Her echocardiographic features changed in accordance with the hemodynamic alterations as treatment progressed. During a routine echocardiography before surgery, a 9-year-old girl with ASD was unexpectedly diagnosed with CP. This explains why her symptoms and signs were inconsistent with isolated ASD, including lower limb edema and hypoalbuminemia caused by impaired diastolic ventricular function. Contrast-enhanced computed tomography confirmed the diagnosis of CP and identified a right ventricular diverticulum. To minimize surgical trauma, a step-by-step surgical approach was adopted. Complete pericardiectomy without cardiopulmonary bypass was first performed to alleviate the pathophysiological abnormalities of CP, followed by percutaneous ASD closure to reduce the excessive right heart volume overload. The diverticulum was not treated because of its small size. The patient had an uneventful postoperative recovery.

## 1 Introduction

Constrictive pericarditis (CP) is a serious yet rare condition, accounting for approximately 0.5%–2% of all heart diseases and 0.6% of interventions in the cardiovascular surgery department. It is associated with poor diagnosis or misdiagnosis due to its non-specific clinical manifestations (1). The coincidental occurrence of CP in a patient with atrial septal defect (ASD) is a rare condition but may provide insights into certain pathophysiological concepts. Maulik et al. reported the absence of ventricular septal bounce and respiratory variations in mitral and tricuspid valve Doppler inflow velocities on the echocardiogram in a patient with CP and associated ASD (2). Ventricular diverticulum is an extremely rare congenital cardiac anomaly that can be diagnosed during childhood when associated with other congenital cardiac abnormalities, and is often described as a component of Cantrell syndrome (3). Each of these three conditions has characteristic ultrasound features and requires differentiation from various other diseases. This report presents a rare case of the coexistence of diverticulum, ASD, and CP, in which complex and interrelated hemodynamic changes emerged as the therapeutic course progressed.

## 2 Case presentation

This report presents a case of a 9-year-old girl in whom an ASD was incidentally identified by echocardiography 3 years ago. Treatment was deferred because of the small size of the ASD and the absence of clinical symptoms. Two years ago, however, the girl was found to be malnourished, and her exercise tolerance began to decrease, which was suspected to be secondary to ASD, prompting her admission to our hospital for further management. On physical examination, her respiratory rate was 25 breaths/min, blood pressure was 105/62 mmHg, and heart rate was 128 beats per minute (bpm). The apical impulse was localized to the fifth intercostal space at the left midclavicular line. No precordial bulge, abnormal pulsations, heaves, or thrills were noted. Cardiac auscultation revealed a regular rhythm with a grade IV/VI systolic murmur at the left second and third intercostal spaces along the sternal border, without accentuation of the pulmonary component of the second heart sound, and no audible pericardial friction rub. Mild bilateral lower limb edema was observed. Her New York Heart Association (NYHA) functional classification was II. Laboratory testing showed negative tuberculosis antibodies and normal rheumatoid factor and antinuclear antibody levels. Initial investigations revealed a white blood cell count of 4.6  ×  10^9^/L (reference range: 4.3–11.3 × 10^9^/L), B-type natriuretic peptide of 157 pg/mL (reference range: 0–100 pg/mL), total protein of 35.1 g/L (reference range: 65–84 g/L), albumin of 18.6 g/L (reference range: 39–54 g/L), prealbumin of 111 mg/L (reference range: 250–400 mg/L), and *γ*-glutamyl transpeptidase of 42.1 U/L (reference range: 5–19 U/L). A chest X-ray showed an enlarged cardiac silhouette with an elevated cardiac apex, accompanied by increased and blurred pulmonary markings, and flattening of the left costophrenic angle, suggesting increased pulmonary blood flow and a small amount of left pleural effusion.

**Figure 1:**
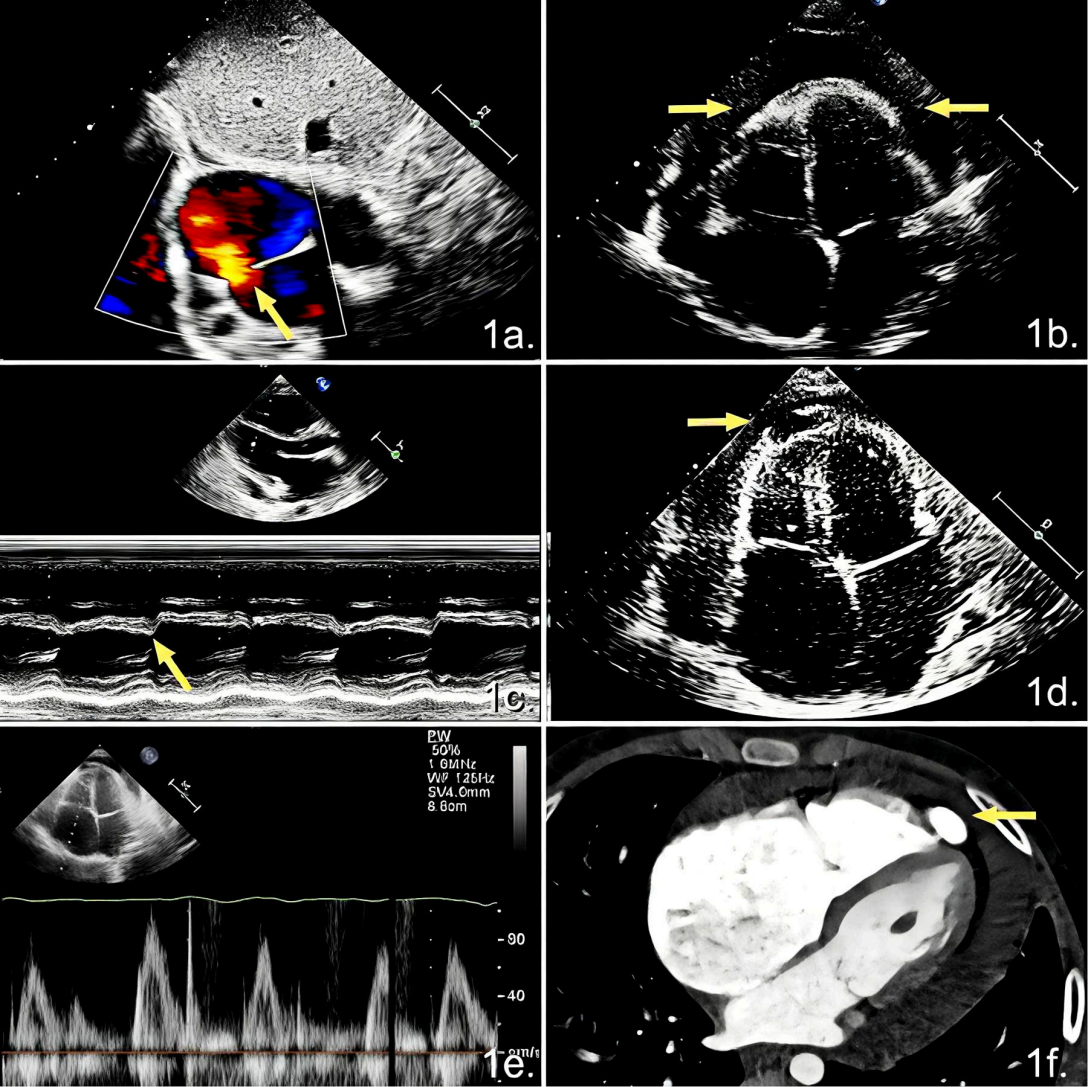
Preoperative TTE and contrast-enhanced cardiac CT images. **(a)** Subcostal bicaval view demonstrating an ASD with a left-to-right shunt. **(b)** Apical four-chamber view showing pericardial thickening associated with pericardial effusion. **(c)** M-mode tracing from the parasternal long-axis view revealing septal bounce in early diastole. **(d)** Apical four-chamber view showing a cystic structure at the right ventricular apex. **(e)** Pulsed-wave Doppler demonstrating >25% respiratory variation in early diastolic tricuspid valve inflow velocity. **(f)** Contrast-enhanced cardiac CT showing a contrast-filled outpouching at the right ventricular apex.

Transthoracic echocardiography (TTE) findings were as follows:

- An ASD with left-to-right shunt measuring 13 mm in diameter (Figure 1a).
- Visceral pericardial thickening (approximately 5.1 mm) was observed around the apical regions of both ventricles and the lateral and posterior walls of the left ventricle (Figure 1b), accompanied by impaired ventricular diastolic filling and an early diastolic septal bounce (Figure 1c), with moderate pericardial effusion.
- A cystic, anechoic area measuring 10.4 × 22.5 mm was identified at the right ventricular apex, without an intraluminal color flow signal (Figure 1d), which was initially considered to be a local pericardial effusion.
- Other TTE findings were marked dilated atria and an enlarged right ventricle. Mild tricuspid regurgitation was observed, with a pulmonary artery systolic pressure of 39 mmHg. The inferior vena cava diameter was 20 mm, with a respiratory collapse rate of <50%. The respiratory variation in early diastolic mitral and tricuspid valve inflow velocities exceeded 25% (Figure 1e).

Ultrasound findings of other organs were hepatic congestion, splenomegaly, 1 cm left pleural effusion, and 3 cm ascites.

Contrast-enhanced cardiac computed tomography confirmed the diagnosis of ASD, demonstrated a visceral pericardial thickening of approximately 5 mm, and showed the contrast agent entering an apical outpouching through a narrow connection to the right ventricular cavity, which was considered a diverticulum (Figure 1f).

The preliminary diagnosis for the girl included congenital heart disease (secundum ASD), CP with moderate pericardial effusion, right ventricular diverticulum, and hypoalbuminemia.

Based on the presence of clinical signs and symptoms of elevated central venous pressure (CVP) and pericardial thickening with impaired diastolic filling, the diagnosis of CP was confirmed. Given the significant pericardial effusion and clinical evidence of CP, manifested by hepatic congestion, splenomegaly, pleural effusion, ascites, hypoalbuminemia, and poor general condition, pericardiectomy via median sternotomy without cardiopulmonary bypass was considered the preferred approach to achieve symptomatic relief while avoiding myocardial injury. The surgical team elected to perform a pericardiectomy as the initial procedure, with planned deferral of ASD repair until the patient's clinical condition improved.

**Figure 2:**
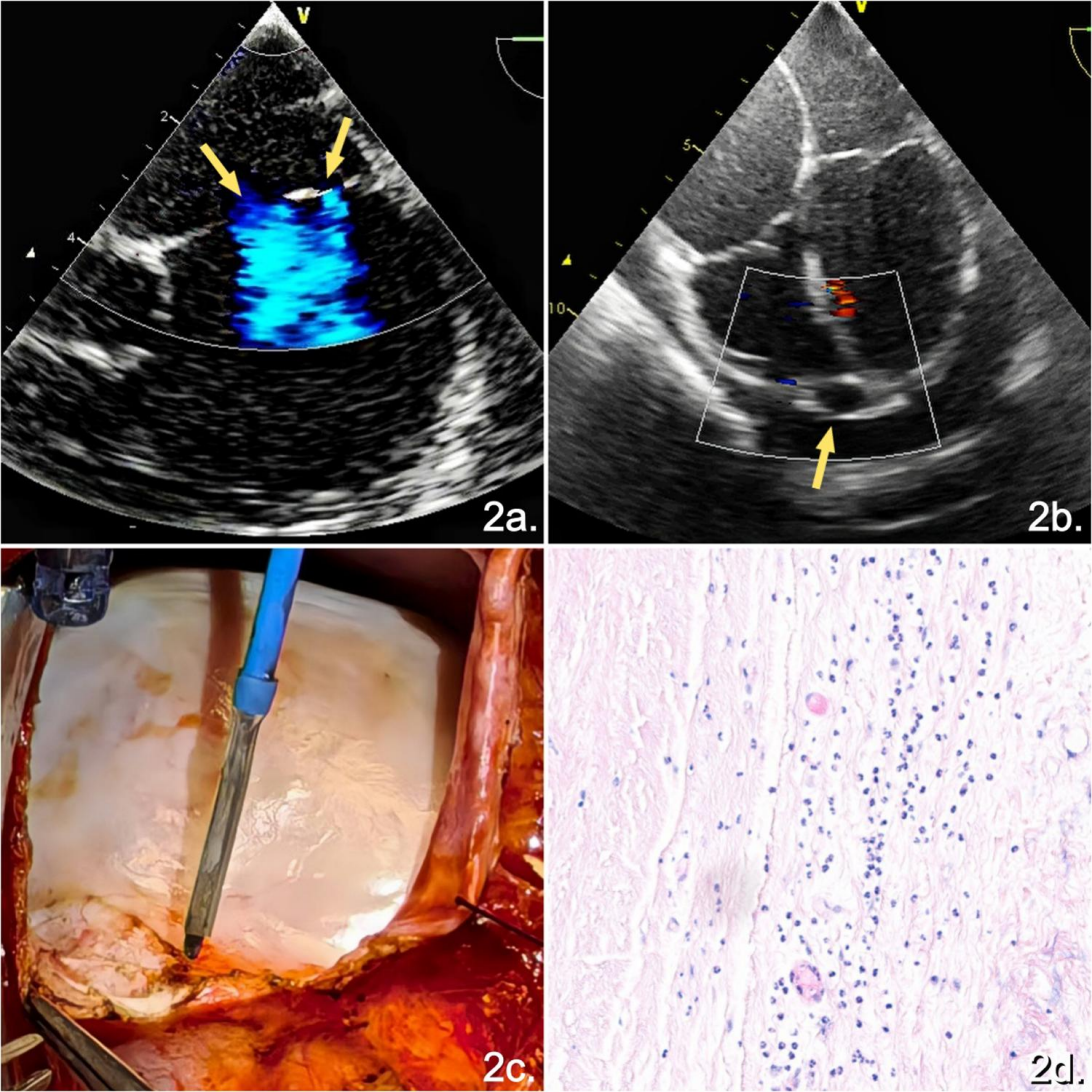
Intraoperative TEE images and pathological findings. **(a)** TEE demonstrating two shunting jets across the atrial septum. **(b)** TEE revealing a cystic structure at the right ventricular apex with no detectable color-flow signal. **(c)** Intraoperative view showing a thickened, adherent, grayish-white pericardium. **(d)** Histopathological examination revealing fibrous tissue hyperplasia with hyaline degeneration (hematoxylin and eosin staining, original magnification ×20).

The patient underwent complete pericardiectomy via median sternotomy under general anesthesia. Intraoperative transesophageal echocardiography (TEE) revealed two defects in the atrial septum, measuring 13 and 3 mm in size, respectively, with a 4 mm distance between them (Figure 2a). Pericardial thickening with moderate effusion was observed, along with a cystic structure at the right ventricular apex, without a detectable intraluminal flow signal (Figure 2b). An intraoperative inspection revealed marked, firm, grayish-white pericardial thickening with adhesions between the visceral and the parietal pericardial layers (Figure 2c). The procedure consisted of complete removal of the anterior pericardium between the phrenic nerves, along with removal of the diaphragmatic pericardium and a portion of the pericardium posterior to both phrenic nerves. The CVP decreased from 25 cmH_2_O (1 cmH_2_O = 0.098kPa) to 15 cmH_2_O following pericardiectomy. A histopathological examination of the resected pericardial tissue confirmed CP with non-specific inflammation (Figure 2d).

Owing to the small size and asymptomatic nature of the diverticulum, the surgical team elected a conservative approach with clinical follow-up.

**Figure 3:**
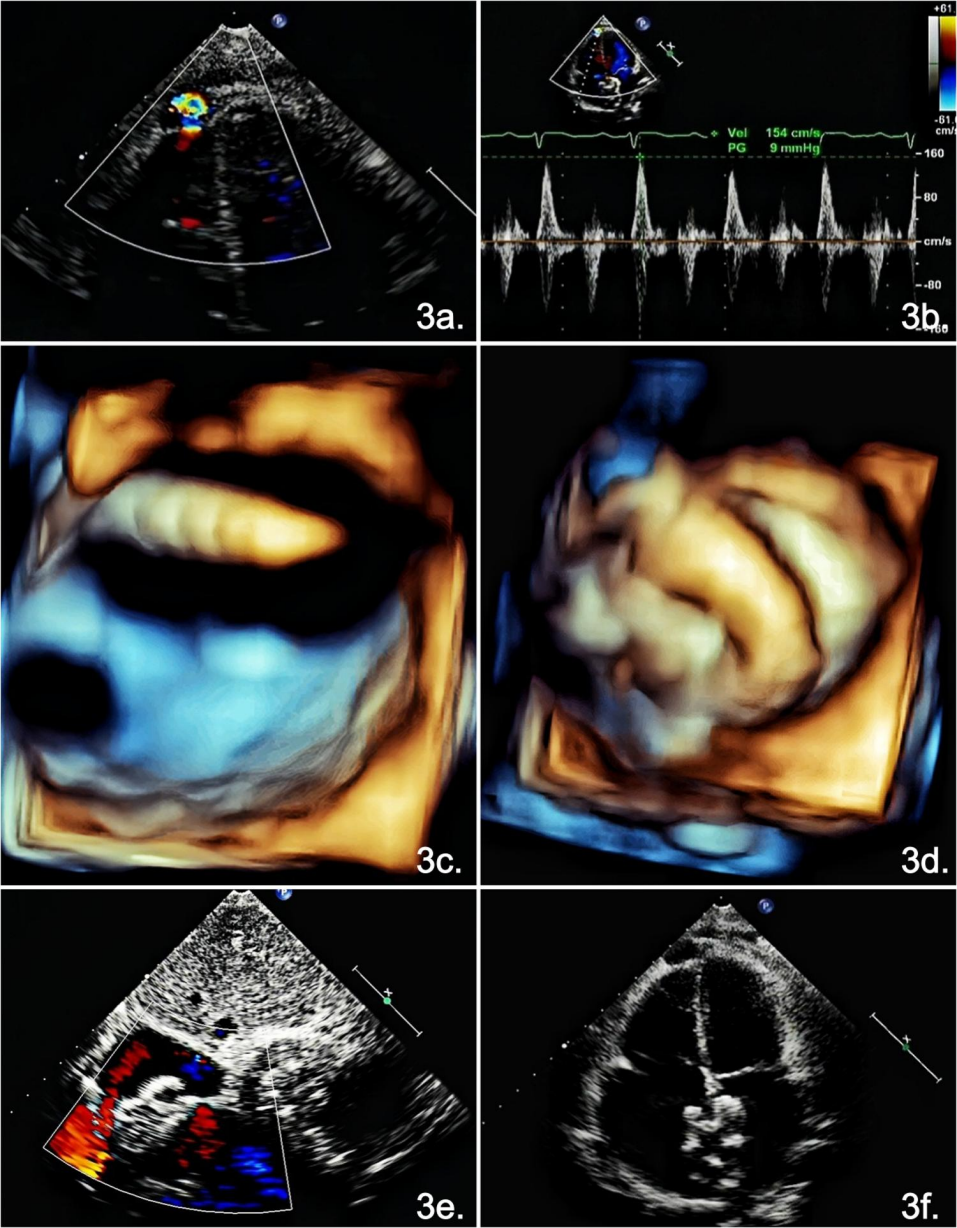
TTE images following pericardiectomy. **(a)** On the first postoperative day, TTE shows communication between the right ventricle and the apical outpouching. **(b)** Spectral Doppler at the neck showing a bidirectional flow signal. **(c)** Three-dimensional TTE showing two defects in the atrial septum, with the delivery sheath advanced through a large defect. **(d)** Three-dimensional TTE confirming complete seating of the double-disc occluder. **(e)** TTE demonstrating a well-positioned occluder without a residual shunt on the eighth postoperative day. **(3f)** Apical four-chamber view showing a persistent diverticulum.

On the first postoperative day, bedside TTE demonstrated normalized ventricular diastolic function and revealed apical outpouching with a 2-mm narrow connection to the right ventricular cavity. Low-velocity bidirectional flow was observed at the neck of the outpouching, indicating that the outpouching was contractile, which was consistent with the hemodynamic features of a diverticulum (Figures 3a,b).

On the second postoperative day, an attempt to wean the patient off mechanical ventilation was unsuccessful, which was attributed to suspected pulmonary edema secondary to left-to-right shunting across the atrial septum. Consequently, an emergency percutaneous ASD occlusion procedure was performed under continuous three-dimensional TTE guidance. Based on the three-dimensional TTE images, specifically the configuration of the two defects, adequate rim tissue, and short interdefect distance, a single double-disc occluder (26 mm, Coyote Type 26, HUAYI Technology Company, China) was selected. Using a femoral venous approach, the delivery sheath was advanced through the large defect into the left atrium. The device was deployed to occlude a large defect and compress an adjacent small defect. Three-dimensional TTE confirmed appropriate device positioning with no residual shunts (Figures 3c,d).

A follow-up TTE on the eighth postoperative day demonstrated no residual interatrial shunt and normal ventricular diastolic function; the diverticulum at the right ventricular apex remained unchanged. The patient was discharged uneventfully (Figures 3e,f).

## 3 Discussion

CP is an inflammatory pericardial disorder frequently triggered by infection, rheumatic disease, tuberculosis, or non-specific inflammation. It is particularly rare in the pediatric population. CP is characterized by pericardial thickening, adhesion, fibrosis, and occasionally calcification, which restrict ventricular diastolic filling (4). Because of its non-specific symptoms, insidious onset, and lack of clear temporal evolution, CP poses a diagnostic challenge. The non-compliant pericardium in CP is responsible for two key pathophysiological phenomena: exaggerated ventricular interdependence and intrathoracic–intracardiac pressure dissociation. Echocardiography is the first-line imaging modality for the diagnosis of CP, although no gold standard exists, despite the proposed Mayo criteria for CP, which include ventricular septal shift and respiratory variation in mitral inflow of ≥14.6% (5).

CP is one of the cardiac causes of protein-losing enteropathy. The markedly elevated systemic venous pressure associated with CP directly obstructs the drainage of intestinal lymph into the thoracic duct, leading to the dilation and rupture of lymphatic vessels and subsequent leakage of protein-rich lymph into the intestinal lumen (6). In the present case, the girl presented with malnutrition 2 years prior to admission, and her serum albumin level was severely reduced upon admission (only 18.6 g/L; reference range: 39∼54 g/L), suggesting that the duration of CP might have been at least 2 years.

In the present case, preoperative TTE revealed pericardial thickening and effusion accompanied by ventricular septal bounce and respiratory variation in mitral inflow. These findings supported the diagnosis of CP; however, the tuberculosis antibody was negative, and there was no history of immunological or rheumatic disorders. Finally, a histopathological examination confirmed CP due to non-specific inflammation. A complete pericardiectomy was performed in this case, which is consistent with recent recommendations from high-volume cardiac centers worldwide advocating complete pericardial resection, including the portion posterior to the left phrenic nerve, pulmonary veins, superior and inferior vena cava, diaphragmatic surface, and both atria (7).

The presence of ASD in a patient with CP may be completely missed on oximetry testing, as the equalization of intracardiac diastolic pressures due to CP can reduce the magnitude of shunting (2). This may explain why the small ASD defect was not detected on preoperative TTE in this case. Following pericardiectomy, an attempt to wean the patient off mechanical ventilation was unsuccessful, which was attributed to pulmonary edema secondary to increased left-to-right shunting across the ASD. Without the restrictive effect of the thickened pericardium, the shunt flow rapidly exacerbated the right ventricular volume overload. Therefore, an emergency percutaneous ASD closure was performed. A fundamental principle in such cases is that multihole secundum ASD should be preferably occluded using a single device (8). In this case, a device 11 mm larger than the maximum diameter of the large defect was selected. The delivery sheath was advanced through the large defect into the left atrium, and the device was deployed to occlude the large defect while compressing the adjacent small defect. Following device deployment, the device position, residual shunt, and potential interference with adjacent cardiac structures were carefully assessed. After ASD closure, the patient was successfully weaned off mechanical ventilation.

Cardiac diverticula are congenital outpouchings that contain all three layers of the cardiac wall (endocardium, myocardium, and epicardium) and exhibit synchronous contractility with the corresponding cardiac chamber, in contrast to aneurysms and pseudoaneurysms, which are akinetic or dyskinetic outpouchings (9). The pathogenic mechanism is postulated to result from failure of the cardiac loop to fuse with the yolk sac during the fourth embryonic week, with these structures subsequently becoming stretched during subsequent phases of embryonic development (10). Bidirectional color-flow Doppler between an extracardiac echo-free space and the ventricular chamber also enables differentiation between simple pericardial effusion and ventricular outpouching (11, 12). In the case of the patient in this study, bidirectional color-flow Doppler was not identified on preoperative TTE and TEE images, resulting in a preoperative misdiagnosis of localized pericardial effusion. However, this finding became evident following the removal of the thickened pericardium.

The management of isolated asymptomatic cardiac diverticula remains controversial. Some authors advocate regular clinical follow-ups and consider surgical intervention unnecessary (13). The surgical team opted for a conservative approach with clinical follow-up in this case, given the small size of the diverticulum and the absence of thrombus.

In this case, both ASD and diverticulum were congenital in origin; however, when CP developed, distinctive and complex ultrasound manifestations appeared, ASD shunt flow was limited, and the diverticulum lacked the characteristic to-and-fro blood flow signal at its neck. The underlying pathophysiology gradually became apparent as treatment progressed, and the staged management strategy ultimately yielded a favorable clinical outcome.

## Data Availability

The original contributions presented in the study are included in this article/Supplementary Material, further inquiries can be directed to the corresponding author.
